# Occurrence Data Sources Matter for Species Distribution Modeling: A Case Study of *Quercus variabilis* Based on Biomod2

**DOI:** 10.1002/ece3.71390

**Published:** 2025-05-08

**Authors:** Yipei Zhao, Jianfeng Liu, Qi Wang, Ruizhi Huang, Wen Nie, Shaowei Yang, Xiangfen Cheng, Maihe Li

**Affiliations:** ^1^ State Key Laboratory of Efficient Production of Forest Resources, Key Laboratory of Tree Breeding and Cultivation of National Forestry and Grassland Administration Research Institute of Forestry, Chinese Academy of Forestry Beijing China; ^2^ Ecology and Nature Conservation Institute Chinese Academy of Forestry Beijing China; ^3^ Swiss Federal Research Institute WSL Birmensdorf Switzerland

**Keywords:** Biomod2, climate change, different occurrence data sources, *Quercus variabilis*, suitable habitats

## Abstract

Climate change is anticipated to escalate the frequency and severity of global natural disasters over the next few decades, thereby significantly reshaping species distributions and populations. Species distribution models (SDMs), as essential tools in biogeography and biodiversity conservation, are pivotal for evaluating the impacts of climate change on species and forecasting their distribution ranges under different climate change scenarios over various periods. However, the absence of necessary background knowledge for model construction significantly affects the accuracy of these models, with the selection of different occurrence data sources being a key factor that constrains the accuracy of model predictions. In this study, using *Quercus variabilis* as a case study, which has diverse ecological, economic, and cultural values, we employed the Biomod2 ensemble modeling platform to comparatively analyze disparities between two different occurrence data sources (i.e., online specimen and scientific survey data) in the species distribution prediction accuracy, relative contribution of major environmental variables, and predicted distribution ranges. Furthermore, we examined potential discrepancies between these two data sources in the migration distance and direction of the species distribution centroid under different future climate scenarios over various periods. Our results indicated substantial differences in the simulation outcomes of SDMs derived from various occurrence data sources. SDMs based on scientific survey data had higher predictive accuracy (AUC = 0.9720, TSS = 0.8370), with the simulated species distribution ranges not only closely matching the actual distributions but also showing more pronounced changes in suitable habitat areas and centroid migration trends under future climate scenarios. In comparison, models based on online specimen data predicted a wider species distribution range, yet exhibited less pronounced trends in suitable area changes and centroid migration under future climate scenarios. Additionally, although the main environmental variables affecting the simulation outcomes from different occurrence data sources were essentially identical, they varied in their contributions and order of importance. Among them, human activity had a relatively stronger contribution for the online specimen data (17.76%), while topographic variables had a stronger impact for the scientific survey data, such as elevation (17.79%). Therefore, the choice of occurrence data sources have a significant impact on SDMs modeling results; this study provides insights and guidance for selecting optimal occurrence data sources to enhance the reliability of SDMs simulations.

## Graphical abstract

This study compares the impact of two data sources—online specimen data and scientific survey data—on the accuracy of Species Distribution Models (SDMs) for *Quercus variabilis* . We found that SDMs based on scientific survey data had higher predictive accuracy and better matched actual distributions, while those based on online specimen data predicted a wider range but showed less pronounced trends under future climate scenarios. The study highlights the importance of selecting optimal data sources to enhance the reliability of SDMs in predicting species distribution under climate change.

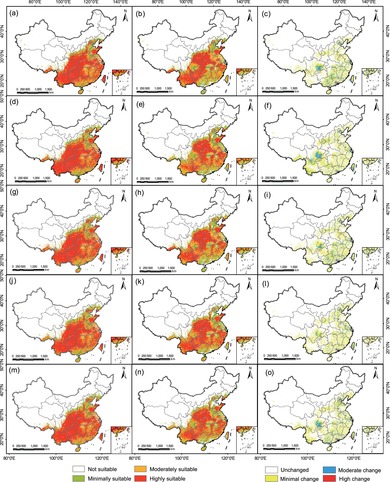

## 1 Introduction

Global climate change is accelerating the degradation, fragmentation, and loss of plants, significantly altering their geographic distribution patterns (Nolan et al. 2018; Feeley and Zuleta 2022; Ma et al. 2023). Numerous studies have shown that many plants are gradually migrating to higher latitudes or altitudes to track more suitable habitats (Sun et al. 2020; Fang et al. 2021; Zhao et al. 2021). Given the profound impact of climate change on plant distribution, it is essential to explore the response of plant distribution patterns to climate change for species conservation and ecosystem restoration (Marks et al. 2016).

Species distribution models (SDMs) are statistical tools for predicting species distribution probabilities based on species occurrence data and corresponding environmental variables (Murphy and Smith 2021; Lu et al. 2024). During modeling, appropriate model algorithms are prioritized according to different modeling purposes, species ecological niche characteristics, and modeling databases (Elith and Leathwick 2009; Wang and Jackson 2023). However, due to differences in principles and algorithms, each model has its own advantages and limitations, and the performance of each model could be unstable as the input data change (Thuiller 2003; Norberg et al. 2019). To improve model prediction accuracy, the Ensemble model (EM), such as Biomod2 (Thuiller et al. 2009) (https://biomodhub.github.io/biomod2/), which integrates the main trends (including mean, median, and percentiles, etc.) and overall changes of multiple models, provides a new approach (Grenouillet et al. 2011; Guisan et al. 2017; Hao et al. 2020). Since its introduction, it has been widely applied due to its flexibility and scalability (Thuiller et al. 2009; Hao et al. 2019).

The development and application of various algorithms has significantly advanced the field of SDMs over the past 30 years, while the quality of species occurrence data remains a critical and easily overlooked constraint on SDMs (Phillips et al. 2009; Beck et al. 2014). Currently, species occurrence data generally consist of species lists, museum records, scientific surveys, and digital online datasets. These datasets mainly include opportunistic, contingent, and semi‐structured citizen science data, as well as systematic, standardized, and well‐structured scientific survey data (Niamir et al. 2016; Fraisl et al. 2022). Of these, the Global Biodiversity Information Facility (GBIF), which is accessed in the form of an online open database, is currently the main source of data for biodiversity research and more than 50% of the data is provided by citizen science (Chandler et al. 2017). Although online open databases compensate for data shortages, they are often challenged by scholars due to their general lack of accuracy and validation (Yesson et al. 2007; Jiménez et al. 2019; Štípková et al. 2024). For example, Tiago et al. (2017) found that the overall performance of SDMs based on citizen science data is lower than that of scientific survey data, mainly attributing this to possible species identification errors due to unprofessional observers, as well as non‐systematic and possible spatial–temporal bias in data collection. Chapman et al. (2024) also reported that the GBIF data cannot capture the latitudinal gradient of species diversity, but rather more closely approximates macroeconomic patterns, since it is often concentrated in places such as densely populated areas, economically developed regions, and nature reserves.

Scientific survey data are collected and processed by professionals on a standardized basis according to the purpose of the research, which ensures the reliability of the data. However, the collection of these data often requires long periods of fieldwork and rigorous standardized methods, which are not only time‐consuming but also costly. This costly and resource‐intensive collection process therefore limits the amount of data available and narrows the coverage of widely geographically distributed data in macroecology. To compensate for this limitation, numerous studies have favored combining citizen science data with scientific survey data as input (Niamir et al. 2011; Zhao et al. 2021; Xian et al. 2023). This capitalizes on the broad coverage of citizen science data and the precision of scientific survey data, but at the same time may mask potential problems with spatial biases in both. These biases arise from a number of sources, such as which level the sampling was conducted (e.g., population or isolated individuals), at what spatial scale the sampling was conducted, and whether the land cover around the sampling site has undergone significant changes. These under‐considered spatial bias factors not only affect the data quality and representativeness but also increase the risk of model overfitting (Boria et al. 2014). Therefore, indiscriminate use of data in combination will lead to increased uncertainty in predictions and reduced model accuracy.

Currently, there is relatively little research addressing the impact of different occurrence data sources on the predictive accuracy of SDMs, and the choice of occurrence data sources for model construction remains under‐investigated. For example, Di Febbraro et al. (2023) integrated citizen science and scientific survey data, revealing the strengths and limitations of the two data sources at different scales and under global change drivers. Their results showed citizen science data significantly improved the predictive ability of biological invasion risks at the national scale, while caution is needed in handling the differences between the two data sources at the regional scale to avoid prediction biases. Similarly, many studies have confirmed that combining expert survey data with semi‐structured citizen science data, which has been manually screened, can improve the predictive accuracy of SDMs (Robinson et al. 2020; Zulian et al. 2021; Stuber et al. 2022). Despite these findings, a recent study on common issues in SDMs modeling revealed that citizen science data contain significant errors. The reasons for these errors include high spatial uncertainty in many early records, specifically manifested as species identification mistakes, and the majority of records lack information on the uncertainty of identification and georeferencing. These errors accumulate, ultimately causing serious distortions and expansions in the inferred relationships between species and their environments, as well as in the suitable distribution areas of species (Soley‐Guardia et al. 2024). Similarly, Lin et al. (2015) used the distribution of moth species in Taiwan as an example to compare the impact of crowd‐sourced data and professionally collected data on the predictive performance of SDMs. The results showed that SDMs built using the GBIF dataset with small sample sizes performed the worst. Given the ongoing debate over the use of different distribution data sources, it is necessary to delve into the impact of different data sources on SDMs prediction results. To clarify the effects of different occurrence data sources on the simulation results of SDMs, we divided the commonly used occurrence data into two categories: online specimen data and scientific survey data. The online specimen data come from commonly used online open datasets, including citizen science and scientific survey data, in which some metadata information is opaque and highly heterogeneous. The scientific survey data originate from organized professional surveys, which have detailed localized information that can respond to the ecological needs and distribution patterns of species.

*Quercus variabilis* , an important dominant species in the temperate and subtropical forests of China, holds significant ecological and economic value (e.g., timber, charcoal, and fungi) (Gao et al. 2018; Han et al. 2022). In China, it plays a key role as an important afforestation and pioneer species and is a key component of carbon sinks in advancing China's carbon neutrality goals (Guo et al. 2024). Hence, this study has selected the ecologically and economically significant *Q. variabilis* as the research subject. Using the Biomod2 modeling platform with two data sources of *Q. variabilis* , our study focuses on the discrepancies in the prediction accuracy under those two occurrence data sources, the primary limiting variables and distribution range, as well as centroid migration distance and direction under future emission scenarios. We specifically test three hypotheses: (1) The prediction results of SDMs based on different occurrence data sources exist significant differences; (2) Across climate scenarios (current and future), predictions based on online specimen data will exhibit higher spatial variability compared to structured scientific surveys; (3) Human‐induced uncertainties in online specimen data will have a stronger impact on model predictions than those in scientific survey data, potentially amplifying projection uncertainties under future climate scenarios. Our study provides a scientific basis for the rational selection of data sources in future SDMs.

## 2 Material and Methods

### 2.1 Species Occurrence Data

**FIGURE 1:**
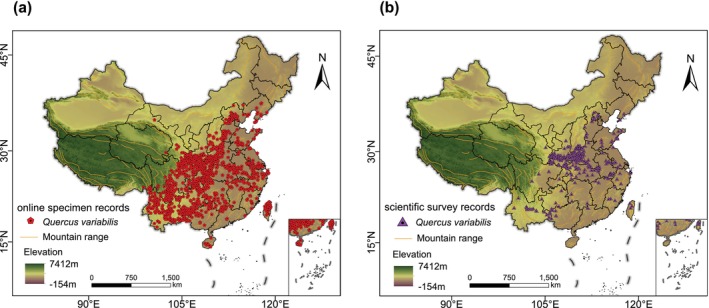
The occurrence records for online specimen data (denoted by pentagons) (a) and scientific survey data (denoted by triangles) (b) after removing the sampling bias and spatial autocorrelation.

Online specimen data mainly originate from GBIF (https://www.gbif.org/), Chinese Virtual Herbarium (CVH) (https://www.cvh.ac.cn/), Teaching Citizen Science Resource Sharing platform (http://mnh.scu.edu.cn/) and China Nature Reserves Biological Specimen Resources Sharing Sub‐platform (http://bhq.papc.cn/). Among them, domestic databases are mainly contributed by herbaria and universities, with well‐preserved specimens with some detailed information. Scientific survey data were obtained from field samples and published literatures. Field sample plots conducted between 2010 and 2023 by our research group (*n* = 426), which were field‐located by GPS and sampling activities were uniformly conducted during the growing season of plants to avoid seasonal bias. Published literatures from 2000 to 2023 (*n* = 411), with reliable sources and precise geographic coordinate records. A total of 4839 and 837 records were obtained for online specimen data and scientific survey data, respectively. To ensure the accuracy of occurrence data from the two data sources, we first removed duplicate coordinates and incomplete information. Subsequently, we excluded records that were located on non‐forested land or that did not align with reality. Finally, to reduce sample bias and the impact of spatial autocorrelation and to improve the predictive performance of our models, we utilized the “Spatially Rarefy Occurrence Data for SDMs” tool in SDMtoolbox (v. 2.5) to retain only one distribution point per each 10 km ×10 km raster (Brown et al. 2017). This tool retains only one occurrence point within the Euclidean distance threshold set by the user, in order to address the issue of model overfitting caused by the spatial clustering of distribution points. Ultimately, after the above filtering, our valid records encompassed 1024 for online specimen data and 243 for scientific survey data (Figure 1).

### 2.2 Preliminary Screening of Environmental Variables

We selected five types of environmental variables to construct SDMs, which are climate variables, soil variables, topographic variables, natural disturbances, and human activity. The climate data were obtained from WorldClim (v. 1.4) (Hijmans et al. 2005; Zhao et al. 2021) at a spatial resolution of 30″, encompassing 19 bioclimatic variables (BIO1‐BIO19) (https://www.worldclim.com/). For the future 2050s (2041–2060) and 2070s (2061–2080) scenarios, we chosed two emission conditions representing the low and high greenhouse gas emission scenarios (RCP2.6 and RCP8.5). These data were derived from the CMIP5 global circulation model, CCSM4 (Community Climate System Model version 4), which is an atmospheric circulation model to simulate the climate in China (Zhao et al. 2021; Peng et al. 2022). CCSM4, released in 2010, shows considerably improved overall model performance compared to previous versions (Gent et al. 2011). It integrates both anthropogenic and natural forcing factors from the 20th century and stands as one of the most effective global climate models (GCMs) for simulating future climate conditions (Abdelaal et al. 2019). Notably, it has the best performance for precipitation simulation and a relatively lower bias over most parts of southwest and northeast China (Yang et al. 2020). Currently, many studies have used CMIP5 CCSM4 to predict the suitable habitats of plants in China, achieving significant outcomes (Zhao et al. 2021; Peng et al. 2022; Geng et al. 2022). Although CMIP6 GCMs have improved in some aspects, CMIP5 GCMs still have higher spatial resolution to date (Geng et al. 2022). Then soil data were derived from the Soil Database of China for Land Surface Modeling with a spatial resolution of 30″ (http://poles.tpdc.ac.cn/zh‐hans/data), consisting of 14 variables representing soil physicochemical properties (Shangguan et al. 2013). Topographic data were derived from the United States Geological Survey 90 m spatial resolution Digital Elevation Model (DEM) data and EarthEnv (https://www.earthenv.org/topography), which provided data on profile curvature (PCUV) and roughness at a 30″ spatial resolution (Amatulli et al. 2018). PCUV measures the rate of change of slope and is considered relevant to ecological processes such as surface water flow velocity, soil moisture, and nutrient cycling (Neteler and Mitasova 2008; Grünewald et al. 2013). Roughness describes the topographic contours and surface heterogeneity. Additionally, the DEM data were processed using ArcGIS (v. 10.2) to obtain slope and aspect information.

Natural disturbances are expressed through the aridity index (AI) and evapotranspiration (ET), obtained from the Global Aridity Index and Potential Evapotranspiration Database (v. 3) (https://figshare.com/articles/dataset/Global_Aridity_Index_and_Potential_Evapotranspiration_ET0_Climate_Database_v2/7504448/6). Here we used the annual average AI and ET, which were calculated from climatic data in WorldClim 2.1 for the years 1970–2000. The AI is the ratio of the total annual precipitation to potential evapotranspiration and is commonly used to measure the degree of aridity in a region (Zomer et al. 2022). According to the generalized climate classification scheme based on AI values, regions with AI < 0.65 are defined as arid areas. Furthermore, given that occurrence data can be influenced by anthropogenic sampling criteria and intensity, we introduced the Global Human Footprint Dataset (HFP) dataset, covering the period 1995–2004, sourced from the Wild Project's 30″ spatial resolution global dataset to assess and quantify potential impacts of human activities on SDMs (https://sedac.ciesin.columbia.edu/data/set/wildareas‐v2‐human‐footprint‐geographic). In total, 41 environmental variables were selected, and their spatial resolution was standardized to 30″, with geographic coordinates in the World Geodetic System 1984.

To reduce the multicollinearity among the environmental variables and test the importance, we used the “raster.cor.matrix” function from the ENMTools (v. 1.1.1) package (Warren et al. 2021) and the “vifstep” function from the usdm (v. 2.1‐7) package (Naimi et al. 2014) in R to calculate the Pearson correlation coefficients and variance inflation factor (VIF) among the variables in the three types of environmental variables, respectively. On this basis, we calculated the relative importance of each environmental variable to the distribution of *Q. variabilis* based on the biomod2 modeling platform (Thuiller 2003), and combined with the Pearson correlation coefficient and VIF, we chose the environmental variables with Pearson less than 0.8, VIF less than 10, and greater relative importance. Through the above processing, 8 bioclimatic variables, 4 topographic variables, 9 soil variables, 1 human activity variable and 1 natural disturbance variable were finally obtained, totaling 23 environmental variables (Table 1).

**TABLE 1:** Environmental variables involve in the prediction modeling of *Q. variabilis* .

Environment variables	Description	Abbreviation	Unit
Climate	Mean Diurnal Range (mean of monthly (max temp—min temp))	Bio2	°C
	Isothermality (BIO2/BIO7) (× 100)	Bio3	—
	Min temperature of the coldest month	Bio6	°C
	Temperature annual range (BIO5‐BIO6)	Bio7	°C
	Mean temperature of the wettest quarter	Bio8	°C
	Precipitation seasonality (coefficient of variation)	Bio15	—
	Precipitation of the warmest quarter	Bio18	mm
	Precipitation of coldest quarter	Bio19	mm
Soil	Exchangeable Na+	NA	me/100 g
	Exchangeable Mg^2+^	MG	me/100 g
	Exchangeable H^+^	H	me/100 g
	Alkali‐hydrolysable N	AN	mg/kg
	Available K	AK	mg/kg
	Clay	CL	g/100 g
	Silt	SI	g/100 g
	pH value (H_2_O)	PH	—
	Soil type	ST	—
Topography	Elevation above sea level	ELEV	m
	Slope	SLOP	°
	Aspect	ASPE	°
	Profile curvature	PCUV	radians/m
Human activity	Human footprint	HFP	%
Natural disturbances	Evapotranspiration	ET	mm

### 2.3 SDMs Construction and Parameter Setting

Assuming that topographic variables, soil variables, human activity, and natural disturbance would remain relatively stable over the next five decades (Fang et al. 2021; Xian et al. 2023), we conducted predictions for the suitable habitat of *Q. variabilis* . To construct SDMs, we utilized the biomod2 package (v. 3.5.1) (Thuiller 2003; Thuiller et al. 2009) in R (v. 4.2.2, R Core Team 2022) with eight algorithms: Generalized Linear Models (GLM), Generalized Boosting Models (GBM), Classification Tree Analysis (CTA), Artificial Neural Networks (ANN), Surface Range Envelop (SRE), Flexible Discriminant Analysis (FDA), Multivariate Adaptive Regression Splines (MARS) and Random Forest (RF). Biomod2 model is a strong learner with high predictive accuracy by combining many weak learners with poor performance, and its simulation accuracy is always higher than that of each single model (Friedman and Popescu 2008). Therefore, in order to make the model easier to run and to strike a good balance between model complexity and prediction performance, we decided to use the default parameter settings (Deka and Morshed 2018). Specifically, for GLM, the default family is binomial with a logit link, and the model formula can be automatically generated or user‐specified. GBM uses a Bernoulli distribution, with 2500 trees, an interaction depth of 7, and a shrinkage rate of 0.001. CTA employs the “class” method and default rpart parameters. ANN employs 5‐fold cross‐validation with a maximum iteration limit of 200. SRE removes the 2.5% quantile of extreme environmental variables. FDA uses the “MARS” method with default settings. MARS automatically generates formulas, with a default penalty of 2 and a backward pruning method. Lastly, RF performs classification with 500 trees, default mtry values, and a nodesize parameter of 5, which specifies the minimum number of samples required to split a node. To enhance the accuracy of simulations and reduce random bias, we generated five sets of pseudo‐absence points (Thuiller et al. 2009). In order to maintain a balance between presence and pseudo‐absence points, we ensured that the number of pseudo‐absence points matched that of the presence points. To assess the average predictive performance of EM, we randomly employed 75% of the sample data as the training set and reserved 25% as the validation set (Fang et al. 2021). Each algorithm was executed five times, resulting in a total of 200 models encompassing the generation of five sets of pseudo‐absence points and five repetitions for each algorithm. The model prediction accuracy was assessed using two metrics: area under the receiver operating characteristic (ROC) Curve (AUC) and True Skill Statistic (TSS)—the values of AUC and TSS are in the range of 0–1. The larger the value of the two, the more accurate the prediction result of the model. In this case, AUC values greater than 0.7 suggest good predictions, while values above 0.9 indicate excellent predictions (Swets 1988). Moreover, TSS values greater than 0.6 suggest good predictions, and values above 0.8 indicate excellent predictions (Allouche et al. 2006; Suicmez and Avci 2023). The associated single model accuracies are presented in the accompanying table (Tables S1 and S2). Here, the models with TSS values greater than 0.7 were selected to construct EM using the weighted average method (WM). In the WM, the distribution results predicted by each individual model are first normalized. Then, the weights between models are calculated based on the ratio of a single model's TSS value to the sum of TSS values of all models. The weights were chosen with reference to Guo et al. (2024), who utilized biomod2 to construct SDMs of major oaks (including *Q. variabilis*) in southern China.

We calculated the importance of each factor in EM, derived the average importance of the variables influencing the species suitability zones, and standardized the average importance values (Arenas‐Castro et al. 2020) (i.e., the factor with the highest ranked importance was assigned an importance value of 1, while the factor with the lowest ranked importance was assigned an importance value of 0).

We employed ArcGIS to transform the model results. The model results were classified to create a gradient classification of suitable habitats, consisting of four levels: unsuitable (0–0.2), minimally suitable (0.2–0.4), moderately suitable (0.4–0.6), and highly suitable (0.6–1) (He et al. 2021). Subsequently, the area of each suitability level was computed based on the grid count corresponding to different suitability levels. To compare the differences in simulation results from different occurrence data sources, we first calculated the absolute difference value between the classified results from different occurrence data sources for the same period. Subsequently, based on the range of these difference values, we categorized the results into four levels: no change (difference = 0), low change (difference = 1), medium change (difference = 2), and high change (difference = 3).

The centroid refers to the distribution center of species within a certain region at a specific moment, and changes in the centroid can reflect the processes of aggregation, dispersion, and migration of a species in spatial distribution during a particular historical stage (Stanton et al. 2012). Therefore, we utilized the ArcGIS plugin SDMtoolbox tool (v. 2.5) to calculate the centroid positions and migration trends of the highly suitable zones of *Q. variabilis* simulated by different occurrence data sources during the current, 2050s, and 2070s periods.

## 3 Result

### 3.1 Model Accuracy Comparison

Under the current climate scenario, we calculated and compared the accuracy of eight individual models and EM using two occurrence data sources. For the online specimen data, GBM and RF exhibited AUC values exceeding 0.90 and TSS values surpassing 0.70. As for the scientific survey data, GLM, GBM, FDA, MARS, and RF demonstrated AUC values exceeding 0.89 and TSS values above 0.70 (Table 2). By comparing the simulation accuracy of EM based on the two occurrence data sources, we observed that all TSS values exceeded 0.75 and AUC values exceeded 0.94 (Table 2), while the accuracy of the simulation based on scientific survey data was slightly higher than that based on online specimen data. Specifically, the AUC value of EM based on scientific survey data was 0.9720, and the TSS value was 0.8370.

**TABLE 2:** Evaluation metrics for individual predictive and integrated models under the current scenario. Performance evaluation metrics for eight individual models and their ensemble models constructed based on different occurrence data sources, including the mean (Mean) and standard deviation (SD) of the area under the curve (AUC) and the true skill statistic (TSS).

Model	AUC				TSS			
	Online specimen		Professional		Online specimen		Professional	
	Mean	SD	Mean	SD	Mean	SD	Mean	SD
GLM	0.8964	0.0154	0.9063	0.0254	0.6812	0.0341	0.7058	0.0534
GBM	0.9108	0.0119	0.9276	0.0195	0.7135	0.0306	0.7312	0.0500
CTA	0.8560	0.0299	0.8556	0.0384	0.6706	0.0383	0.6602	0.0557
ANN	0.8566	0.0289	0.8530	0.0318	0.6438	0.0481	0.6218	0.0599
SRE	0.6824	0.0156	0.6846	0.0434	0.3647	0.0308	0.3690	0.0873
FDA	0.8958	0.0149	0.8952	0.0264	0.6934	0.0305	0.7099	0.0483
MARS	0.8999	0.0117	0.8993	0.0286	0.6966	0.0322	0.7084	0.0605
RF	0.9078	0.0122	0.9331	0.0195	0.7205	0.0318	0.7421	0.0468
EM	0.9480	—	0.9720	—	0.7560	—	0.8370	—

Abbreviations: ANN, artificial neural networks; CTA, classification tree analysis; FDA, flexible discriminant analysis; GBM, generalized boosting models; GLM, generalized linear models; MARS, multivariate adaptive regression splines; RF, random forest; SRE, surface range envelop.

### 3.2 Comparison of Dominant Environmental Variables Under the Current Scenario

**FIGURE 2:**
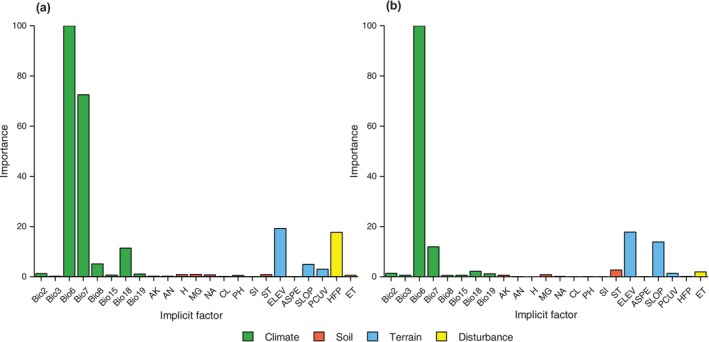
Importance of influence variables affecting the distribution of *Quercus variabilis* suitable areas in different occurrence data sources under the current scenario: (a) online specimen data and (b) scientific survey data. The influence variables are shown in Table 1. (Bio2: mean diurnal range; Bio3: isothermality; Bio6: min temperature of the coldest month; Bio7: temperature annual range; Bio8: mean temperature of the wettest quarter; Bio15: precipitation seasonality; Bio18: precipitation of the warmest quarter; Bio19: precipitation of coldest quarter; NA: exchangeable Na^+^; MG: exchangeable Mg^2+^; H: exchangeable H^+^; AN: alkali‐hydrolysable N; AK: available K; CL: clay; SI: silt; PH: PH value; ST: soil type; ELEV: elevation above sea level; SLOP: slope; ASPE: aspect; PCUV: profile curvature; HFP: human footprint; ET: evapotranspiration).

**FIGURE 3:**
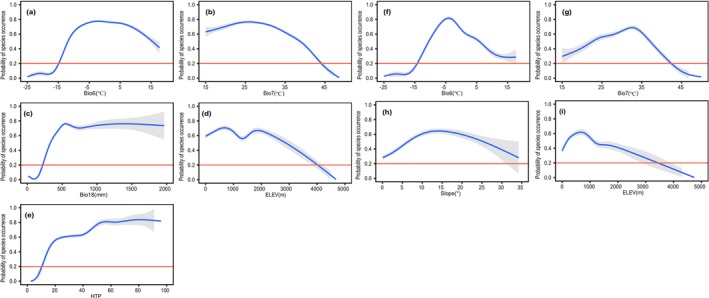
EM‐based response curves represent the relationship between the probability of presence of *Quercus variabilis* and the major impact variables in different occurrence data sources in the current scenario. Where, (a–e) is the response curve of online specimen data, and (f–h) is the response curve of scientific survey data. (a) Bio6; (b) Bio7; (c) Bio18; (d) Elev; (e) HFP; (f) Bio6; (g) Bio7; (h) SLOP; (i) ELEV.

In response to the magnitude of the contribution of environmental variables in the two occurrence data sources, environmental variables with a contribution of more than 10% were selected as the main environmental variables for analysis in this study. Generally, under the current climate scenario, the simulation results of the two occurrence data sources indicated that climatic and topographic variables were the main environmental variables affecting species distribution (Figure 2). Notably, human activity (i.e., HFP) was also considered as the main environmental variable in the online specimen data. Additionally, we found that although Bio6 showed the strongest contribution across occurrence data sources, there were differences in the contribution of other major variables and their order of importance. Specifically, for online specimen data, the main environmental variables in decreasing order of contribution were Bio6 (100%), Bio7 (72.50%), ELEV (19.20%), HFP (17.76%), and Bio18 (11.43%). For the scientific survey data, the main environmental variables in decreasing order of contribution were Bio6 (100%), ELEV (17.79%), SLOP (13.87%), and Bio7 (11.95%). Among them, Bio7, Bio18, and HFP all contributed much more to the online specimen data than to the scientific survey data, while ELEV and SLOP both ranked higher in importance than the online specimen data. Furthermore, response curves based on the probability of species presence in relation to the main environmental variables showed that the main environmental variables in the scientific survey data exhibited more significant trends, and their curves had steeper peaks (Figure 3).

### 3.3 Comparison of *Q. variabilis* Habitat Suitability Areas Under Different Scenarios

**FIGURE 4:**
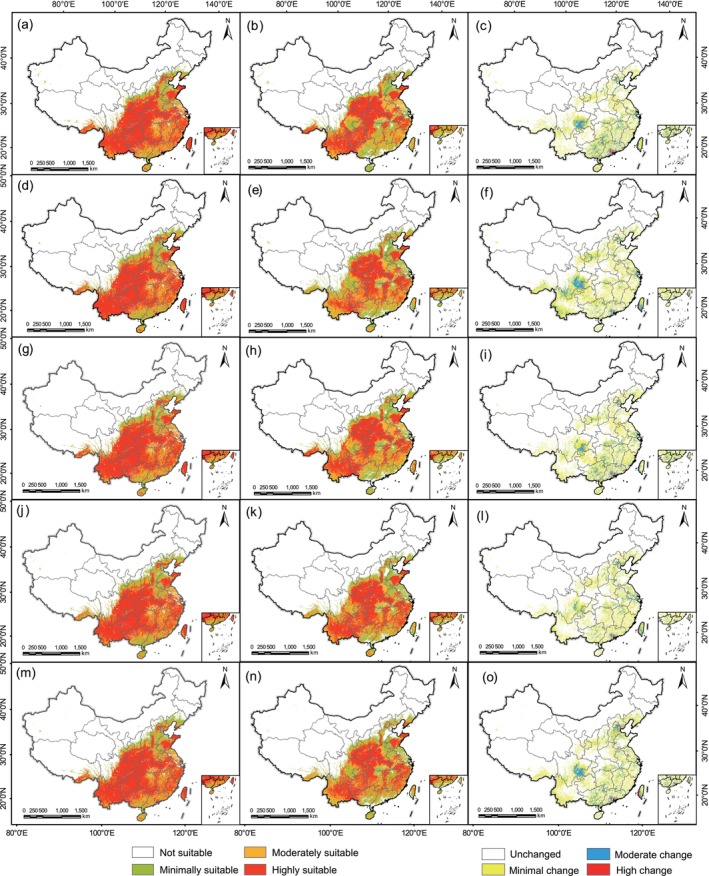
The suitable distribution areas of *Quercus variabilis* simulated by different occurrence data sources for the current, 2050s, and 2070s. In this figure, (a–c) represent the current scenario, (d–f) represent the RCP2.6 scenario in the 2050s, (g–i) represent the RCP8.5 scenario in the 2050s, (j–l) represent the RCP2.6 scenario in the 2070s, and (m–o) represent the RCP8.5 scenario in the 2070s. The first column displays simulation results based on online specimen data, the second column displays results based on scientific survey data, and the third column illustrates the differences in simulation results between the two occurrence data sources.

Generally, the SDMs simulation results indicated that there were varying degrees of discrepancies in the modeling of *Q. variabilis* 's suitable distribution areas between different occurrence data sources under different climate scenarios (Figure 4). Specifically, for the discrepancies in the extent of suitable distribution areas, under different climate scenarios, the regions with the high change were primarily located in the Pearl River Delta region and the plains of southwestern Taiwan, where the online specimen data modeled a highly suitable distribution area, while the scientific survey data modeled an unsuitable distribution area. The region of moderate change was mainly located in the Sichuan Basin, where the online specimen data modeled a highly suitable level, while the scientific survey data modeled a minimally and moderately suitable level. The area with the minimal change was the most widely distributed, covering basically the entire suitable distribution area of the species. Additionally, the unchanged area was mainly located in the area surrounded by Wushan Mountain, Dabashan Mountain, Qinling Mountain, and Dabie Mountain, and the simulation results from different occurrence data sources were all highly suitable distribution areas.

For the differences in trends in suitable distribution areas, the trends in suitable distribution areas modeled by the scientific survey data were more significant under different future climate scenarios. Specifically, simulation results from scientific survey data indicated that the trends in changes of suitable distribution areas for *Q. variabilis* are more pronounced under different climate scenarios. Specifically, in the future, under the RCP2.6 scenario for the 2050s, the highly suitable habitats in the Yunnan‐Guizhou Plateau and Sichuan Basin are expected to contract, while those in Hunan, Jiangxi, Zhejiang, and Fujian will show a significant expansion trend. Additionally, the moderately suitable habitats in the North China Plain are expected to contract and gradually transition to minimally suitable habitats (Figure 4e). By the 2070s, the expansion of highly suitable habitats in the Yunnan‐Guizhou Plateau is anticipated, while the contraction trend of moderately suitable habitats in the North China Plain will become more evident (Figure 4k). Under the RCP8.5 scenario for the 2050s, the highly suitable habitats in the Yunnan‐Guizhou Plateau are predicted to contract further (Figure 4h), with a significant reduction by the 2070s (Figure 4n). In contrast, the expansion of highly suitable habitats in coastal areas such as Fujian and Zhejiang will be most pronounced, and the trend of contraction in the moderately suitable habitats of the North China Plain in the 2070s will also be particularly notable (Figure 4n). In contrast, *Q. variabilis* 's suitable distribution area based on online specimen data did not show a significant trend under different climatic scenarios.

**FIGURE 5:**
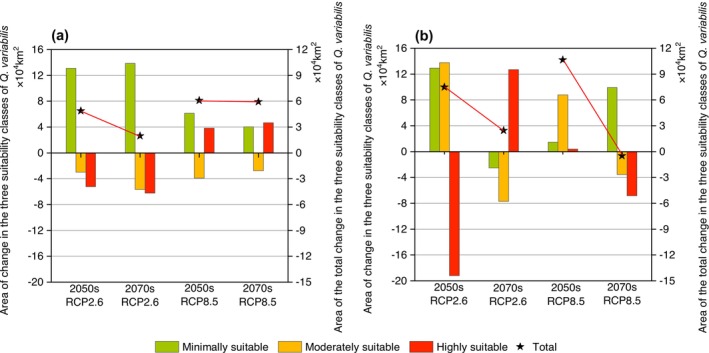
Changes in the area of suitable areas for *Quercus variabilis* under different future climate scenarios simulated by different occurrence data sources, including minimally, moderately, and highly suitable distribution areas: (a) Online specimen data and (b) scientific survey data. The left *Y*‐axis is the change in the area of suitable area of different occurrence data sources, and the right *Y*‐axis is the change in its total area. In the figure, the pentagram represents the total area of the species' suitable distribution.

For differences in the area of suitable distribution regions, compared to current scenario simulations, the total area of suitable habitats for *Q. variabilis* , as predicted by diverse occurrence data sources, exhibited a rising trend under future climate scenarios. Notably, both occurrence data sources exhibited the most significant increase in total suitable area under the RCP8.5 scenarios in the 2050s, reaching 6.07 × 10^4^ km^2^ and 10.64 × 10^4^ km^2^, respectively. However, significant disparities emerged among the areas of different suitability regions (Figure 5). Specifically, the simulation results from online specimen data indicated that the area of minimally suitable habitats for *Q. variabilis* changed most notably, displaying a consistent trend of increase, especially under the RCP2.6 scenario. Meanwhile, the area of moderately suitable habitats generally exhibited a decreasing trend. In terms of highly suitable habitat areas, there was a decrease under the RCP2.6 scenario and an increase under the RCP8.5 scenario (Figure 5a). However, compared to the simulation results from online specimen data, the simulations based on scientific survey data indicated that the overall change in the area of minimally suitable habitats for *Q. variabilis* was smaller, while the variation in the area of highly suitable habitats was most significant. Particularly, under the RCP2.6 scenario, the area of highly suitable habitats decreased the most by the 2050s, reaching 19.20 × 10^4^ km^2^, and increased the most by the 2070s, reaching 12.68 × 10^4^ km^2^ (Figure 5b).

**FIGURE 6:**
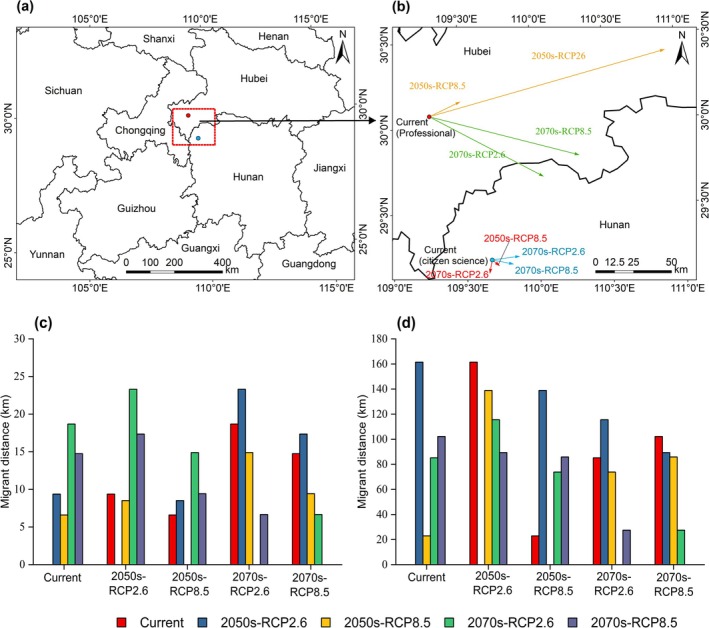
Migration of *Quercus variabilis* high suitable habitat centers under different periods of climatic conditions: (a) shows the centroid under the current climate scenario, with red dots representing the results of scientific surveys and blue dots indicating the results of online specimen data; (b) illustrates the migration routes under future climate scenarios, while (c) and (d) respectively present the predicted migration distances based on online specimen data and scientific survey data.

### 3.4 Comparison on Centroid Migration of Highly Suitable Habitats Under Different Occurrence Data Sources

There were significant differences between two occurrence data sources in the centroid and its migration trends of the highly suitable distribution areas for *Q. variabilis* , where the centroid migrations simulated from scientific survey data being more extensive(Figure 6). Specifically, based on online specimen data simulations, the current centroid of the highly suitable area for *Q. variabilis* was located in Longshan County, Xiangxi Tujia and Miao Autonomous Prefecture, Hunan Province (109.6722°E, 29.2148°N) (Figure 6b). Under future climate scenarios, the centroid showed a trend of moving eastward, with migrations of 9.37 and 6.59 km under the RCP2.6 and RCP8.5 scenarios for the 2050s, respectively; and 18.68 and 14.75 km under the RCP2.6 and RCP8.5 scenarios for the 2070s, respectively (Figure 6c). However, for scientific survey data simulations, currently, the centroid of the highly suitable area for *Q. variabilis* was located in Enshi Tujia and Miao Autonomous Prefecture, Hubei Province (109.2842°E, 30.0705°N) (Figure 6b). Under future climate scenarios, the centroid also showed a trend of moving eastward, with migrations of 161.44 and 22.91 km under the RCP2.6 and RCP8.5 scenarios for the 2050s, respectively; and 85.18 km and 102.12 km under the RCP2.6 and RCP8.5 scenarios for the 2070s, respectively (Figure 6d).

## 4 Discussion

The impact of different occurrence data sources on the accuracy of SDMs simulations exhibited significant variations, primarily determined by the criteria, intensity, and biases of the data sampling process (Syfert et al. 2013; Frey et al. 2013; Inman et al. 2021). The sampling criteria were always set according to research objectives and ecological characteristics of the species studied. In particular, whether the data originate from areas of continuous natural forest with minimal human disturbance could significantly affect the accuracy of model simulations. However, online specimen data, which integrate nationally funded research records and crowdsourced platforms such as iNaturalist, exhibited heterogeneous sampling protocols that hinder standardization and provenance tracking, thereby introducing inherent uncertainties in SDMs outputs (Yesson et al. 2007). Citizen science data, on the other hand, were typically collected under a wide range of sampling conditions by different levels of observers over a wide range of spatial scales and lack standardized sampling processes (Yesson et al. 2007; Sterner and Franz 2017). In contrast, scientific survey data, mostly derived from areas with less human disturbance, contained a richer information content although less in sample quantity, effectively reducing the model's uncertainty (Robinson et al. 2020). For example, Beck et al. (2013) found that, despite the GBIF provided a vast amount of species distribution data, the information content was relatively low; whereas independently compiled data could comprehensively cover the species' climatic distribution areas, greatly contributing to the identification of species distribution areas. Qian et al. (2018) found that there was a large bias in analyses based on GBIF data exploring the correlation between species richness and climate variables. Sample size may similarly affect the modeling accuracy of SDMs (Wang and Jackson 2023). According to the findings of Stockwell and Peterson (2002), larger sample sizes may contain more erroneous or low‐quality samples, which may reduce model accuracy and increase the risk of overfitting. Therefore, SDMs based on larger sample sizes of online specimen data may have relatively lower modeling accuracy.

Sampling bias often stemed from incomplete or excessive sampling, allowing for unquantifiable heterogeneity in spatial sampling intensity (Newbold 2010; Kramer‐Schadt et al. 2013). Such biases included mainly temporal, spatial, environmental, and taxonomic errors (Soberón et al. 2000), and were common in opportunistic data lacking systematic sampling efforts, including incidental records at specific locations for species (e.g., museum and herbarium records) or observations reported by the public (Hefley et al. 2013). As Elith et al. (2006) found, museum data were often collected without planning, and the intention and methods of collection were poorly understood, so random or stratified samples lacking systematic planning often led to sampling bias. Graham et al. (2004) showed that museum data were often subject to high levels of inaccuracy, and were largely due to incorrect species identification, ambiguous recording of distribution points, differences in locational coordinate systems, and insufficient accuracy of maps used for location. Additionally, data from museums and herbaria typically originated from areas with high accessibility, such as those near roads and villages (Phillips et al. 2009). These areas were frequently subject to strong human disturbance, leading to inevitable oversampling (Kadmon et al. 2004; Pearce and Boyce 2006). For citizen science data, there may be greater uncertainty due to the diversity of sources and non‐standardized methods (Yesson et al. 2007). Therefore, the arbitrary compilation of such data frequently served as the data source for SDMs, introducing substantial bias into model construction and affecting the accuracy of model predictions. Furthermore, the increase in sampling means has made access to citizen science data easier, but has exacerbated the overrepresentation of certain areas within the study area, which can lead to significant spatial bias in the occurrence data collected. However, sampling bias in species occurrence records was ubiquitous. When integrating datasets from different sampling schemes, the weighting of environmental covariates in models can be distorted, leading to potential overfitting of sampling bias rather than genuine species‐environment relationships, particularly for widespread species (Phillips et al. 2009; Yackulic et al. 2013). To mitigate such bias, Phillips et al. (2009) proposed using background data with sampling bias congruent with occurrence data, such as incorporating all distribution records of target‐groups as background, a method proven to substantially improve model accuracy under strong bias scenarios. Fourcade et al. (2014) further demonstrated that systematic sampling strategies (e.g., retaining a single record per grid cell) effectively reduce bias impacts and outperform complex correction approaches. Additionally, Xu et al. (2024) quantified and corrected the sample bias by quantifying the clustering degree of sample points in geographical and environmental feature spaces, and selecting the background points that have a high clustering degree with the sample points. This provided a new idea for solving the data bias problem. Notably, correction efficacy was jointly constrained by bias types (e.g., geographic bias, sampling gaps at distribution edges) and species traits (e.g., niche breadth) (Støa et al. 2018), necessitating adaptive strategy selection based on modeling objectives (spatial distribution prediction vs. elucidating ecological mechanisms). In conclusion, the choice of methods for correcting distribution point bias remained a focal point and challenge for future research. Based on the findings of this study, when selecting occurrence records, we should prioritize scientific survey data that is complete and high‐precision in order to minimize the impact of human factors.

In this study, SDMs constructed from different occurrence data sources showed significant differences in species distribution ranges, environmental factor contributions, response curves, centroid migration, and future trends. In particular, there were large biases in the prediction of species ranges in the Sichuan Basin, the Pearl River Delta, and the plains of southwestern Taiwan. The study by Fang et al. (2011) indicated that the Sichuan Basin was not a suitable distribution area for *Q. variabilis* , which contradicted the modeling results of the online specimen data. This bias may be due to over‐sampling or misidentification of species in these areas, resulting in an overestimation of the suitable distribution level of the species in these areas. Also, the low‐lying topography and high rainfall of the Sichuan Basin, which is susceptible to flooding, combined with the high sensitivity of *Q. variabilis* to inundation (Yi et al. 2006), may limit its natural distribution in this region. Additionally, the wide spatial distribution of the online specimen data may have exaggerated the species' adaptation to climate change. This can result in wider response curves, smoother and smaller trends in suitable areas under future climate change, and less pronounced centroid migration—all of which may deviate the results from the true value. Comparatively, the scientific survey data were mainly collected from the hotspots of species distribution, such as the Qinling Mountains, the Taihang Mountains, and the Wushan Mountains, which follow a standardized sampling process and can more accurately reflect the distribution characteristics of *Q. variabilis* (Castellanos et al. 2019). Additionally, the simulation results were more consistent with the distribution range of *Q. variabilis* in the Atlas of Woody Plants in China (Fang et al. 2011).

Existing studies employing different modeling algorithms and distribution data sources have systematically analyzed the spatial distribution patterns of *Q. variabilis* , identifying key environmental drivers. Our results demonstrated that the temperature variable bio6 consistently showed the highest contribution across datasets, aligning with findings by Gao et al. (2016) at the East Asian regional scale. This indicated winter extreme low temperature as the decisive factor limiting its suitable habitat, with its sun‐loving and drought‐tolerant traits further exacerbating sensitivity to temperature stress (Flora of China Editorial Committee 1999). Furthermore, since *Q. variabilis* primarily inhabits humid and semi‐humid regions, precipitation may not constitute a primary limiting factor. However, Chen et al. (2022) reported the highest contribution from bio12, creating a notable discrepancy. This divergence likely stemed from the species' broad ecological amplitude spanning warm‐temperate to subtropical zones, where geographical selection of distribution points critically influences species–environment relationship interpretation. Then the topographic variable had a relatively higher order of contribution in the SDMs constructed from the scientific survey data, suggesting that the distributional characteristics of *Q. variabilis* preferring complex mountainous topography were taken into account during sampling. Meanwhile, HFP had a significant impact on the simulation results based on online specimen data, which was closely related to its collection mode mainly from areas with intensive human activities. This was further confirmed by studies such as Newbold (2010), who found a significant positive correlation between sampling density and GDP and human impact, suggesting that online specimen data are unevenly distributed and subject to greater human interference. This finding was consistent with the conclusions of this study. Overall, blindly integrating data with high heterogeneity can reduce the accuracy of model simulations. On the contrary, scientific survey data can be used as the preferred data source for the construction of SDMs due to its structure, standardization, and representativeness, which reduces human interference and thus provides more accurate simulation results.

Different GCMs introduced uncertainty into SDMs predictions, which may affect distribution data sources selection (Peng et al. 2022). To enhance robustness, Guo et al. (2024) combined data from 11 GCMs and used a voting method to predict the distribution of oaks in southern China. Therefore, future research should consider applying multiple GCMs to more accurately assess the potential impacts of climate change on species distribution. Additionally, compared with CMIP5, CMIP6 considered socio‐economic factors and complex physical processes, yielding results closer to observations (Wang, Li, et al. 2022; Wang, Lv, et al. 2022). Therefore, we will combine CMIP6 data for further verification in future work.

Species distribution was typically influenced by a multitude of environmental factors; considering only climatic factors can lead to predictive biases (Sheth et al. 2020). Studies have shown that the exclusion of variables related to human activities can significantly reduce the predictive performance of SDMs (Frans and Liu 2024). However, due to limitations in data availability, a large number of studies usually assumed that these variables will remain stable over the coming decades, especially in aspects such as land use, building area, and human footprint index (Frans and Liu 2024). This study was also subject to this assumption. To construct more rational SDMs, it is necessary to develop more complex models that can effectively integrate or replace such environmental data, which also provides an important direction for the future optimization and development of models.

## 5 Conclusions

This study conducted a comparative analysis on the differences in the suitable habitat areas of *Q. variabilis* and its main limiting variables identified between two different occurrence data sources using the EM, and explored the variations in the trends of suitable habitat areas under different climate scenarios. The results demonstrated that standardized, structured, and well‐organized scientific survey data yielded higher simulation accuracy and more reliable outcomes. In contrast, online specimen data, which comprise various sources and are subject to significant human‐related biases, showed lower accuracy in simulations. Although the main environmental variables affecting the simulation outcomes from different occurrence data sources were consistent, significant differences were observed in their contributions and order of importance. Among them, the contribution of human activity variables to the simulation results based on online specimen data was relatively higher, while the contribution of topographical variables to the simulation results based on scientific survey data was relatively higher. Furthermore, the changes in the area, migration distance, and changes in the species probability of presence response curve under different future climate scenarios were less sensitive for online specimen data. Therefore, scientific survey data should be recommended as the primary source for SDMs, with online specimen data providing supplemental information. The findings of this study offer a scientific theoretical basis and practical recommendations for the selection of species occurrence data sources in models, contributing to the enhanced reliability of SDMs simulations.

## Author Contributions

**Yipei Zhao:** data curation (equal), investigation (equal), methodology (equal), resources (equal), software (lead), writing – original draft (equal). **Jianfeng Liu:** funding acquisition (lead), supervision (lead),methodology (equal), resources (equal), writing – original draft (equal), writing – review and editing (equal). **Qi Wang:** investigation (equal), writing – review and editing (equal). **Ruizhi Huang:** resources (equal), validation (equal), visualization (equal), writing – review and editing (equal). **Wen Nie:** methodology (equal), software (equal), writing – review and editing (equal). **Shaowei Yang:** investigation (equal), software (equal), writing – review and editing (equal). **Xiangfen Cheng:** investigation (equal), writing – review and editing (equal). **Maihe Li:** supervision (equal), writing – original draft (equal), writing – review and editing (equal).

## Conflicts of Interest

The authors declare no conflicts of interest.

## Supporting information


**Table S1.** Accuracy metrics (AUC and TSS) of individual species distribution models based on online specimen data. Model refers to the specific individual model, Run indicates the number of model runs, and PA denotes the number of pseudo‐absence replicates.
**Table S2.** Accuracy metrics (AUC and TSS) of individual species distribution models based on scientific survey data. Model refers to the specific individual model, Run indicates the number of model runs, and PA denotes the number of pseudo‐absence replicates.

## Data Availability

The data used in this paper, including environmental variables and species occurrence records, is available on the Open Science Framework. DOI: https://doi.org/10.17605/OSF.IO/4FP8J
