# Supplementary material for: Occurrence Data Sources Matter for Species Distribution Modeling: A Case Study of Quercus variabilis Based on Biomod2
Source: Ecol Evol. 2025 May 8;15(5):e71390. doi: 10.1002/ece3.71390 (PMC12061470; doi:10.1002/ece3.71390)
Supplement: Supplementary file 1 — Table S1. Accuracy metrics (AUC and TSS) of individual species distribution models based on online specimen data. Model refers to the specific individual model, Run indicates the number of model runs, and PA denotes the number of pseudo‐absence replicates. Table S2. Accuracy metrics (AUC and TSS) of individual species distribution models based on scientific survey data. Model refers to the specific individual model, Run indicates the number of model runs, and PA denotes the number of pseudo‐absence replicates. [file ECE3-15-e71390-s001.doc]

**Table S1. Accuracy metrics (AUC and TSS) of individual species distribution models based on online specimen data. Model refers to the specific individual model, Run indicates the number of model runs, and PA denotes the number of pseudo-absence replicates.**

| Model | Run | PA | AUC | TSS |
| --- | --- | --- | --- | --- |
| GLM | RUN1 | PA1 | 0.907 | 0.685 |
| GLM | RUN1 | PA2 | 0.892 | 0.713 |
| GLM | RUN1 | PA3 | 0.895 | 0.694 |
| GLM | RUN1 | PA4 | 0.894 | 0.653 |
| GLM | RUN1 | PA5 | 0.914 | 0.723 |
| GLM | RUN2 | PA1 | 0.898 | 0.669 |
| GLM | RUN2 | PA2 | 0.875 | 0.66 |
| GLM | RUN2 | PA3 | 0.884 | 0.661 |
| GLM | RUN2 | PA4 | 0.888 | 0.649 |
| GLM | RUN2 | PA5 | 0.899 | 0.739 |
| GLM | RUN3 | PA1 | 0.902 | 0.673 |
| GLM | RUN3 | PA2 | 0.903 | 0.709 |
| GLM | RUN3 | PA3 | 0.896 | 0.698 |
| GLM | RUN3 | PA4 | 0.885 | 0.653 |
| GLM | RUN3 | PA5 | 0.871 | 0.655 |
| GLM | RUN4 | PA1 | 0.872 | 0.629 |
| GLM | RUN4 | PA2 | 0.916 | 0.717 |
| GLM | RUN4 | PA3 | 0.914 | 0.706 |
| GLM | RUN4 | PA4 | 0.88 | 0.657 |
| GLM | RUN4 | PA5 | 0.919 | 0.718 |
| GLM | RUN5 | PA1 | 0.931 | 0.71 |
| GLM | RUN5 | PA2 | 0.884 | 0.697 |
| GLM | RUN5 | PA3 | 0.91 | 0.718 |
| GLM | RUN5 | PA4 | 0.893 | 0.617 |
| GLM | RUN5 | PA5 | 0.887 | 0.626 |
| GBM | RUN1 | PA1 | 0.924 | 0.714 |
| GBM | RUN1 | PA2 | 0.91 | 0.721 |
| GBM | RUN1 | PA3 | 0.9 | 0.706 |
| GBM | RUN1 | PA4 | 0.907 | 0.669 |
| GBM | RUN1 | PA5 | 0.927 | 0.751 |
| GBM | RUN2 | PA1 | 0.916 | 0.722 |
| GBM | RUN2 | PA2 | 0.911 | 0.737 |
| GBM | RUN2 | PA3 | 0.906 | 0.722 |
| GBM | RUN2 | PA4 | 0.897 | 0.69 |
| GBM | RUN2 | PA5 | 0.907 | 0.767 |
| GBM | RUN3 | PA1 | 0.916 | 0.726 |
| GBM | RUN3 | PA2 | 0.911 | 0.741 |
| GBM | RUN3 | PA3 | 0.919 | 0.726 |
| GBM | RUN3 | PA4 | 0.891 | 0.657 |
| GBM | RUN3 | PA5 | 0.903 | 0.695 |
| GBM | RUN4 | PA1 | 0.89 | 0.665 |
| GBM | RUN4 | PA2 | 0.924 | 0.733 |
| GBM | RUN4 | PA3 | 0.934 | 0.762 |
| GBM | RUN4 | PA4 | 0.903 | 0.69 |
| GBM | RUN4 | PA5 | 0.928 | 0.727 |
| GBM | RUN5 | PA1 | 0.926 | 0.742 |
| GBM | RUN5 | PA2 | 0.9 | 0.721 |
| GBM | RUN5 | PA3 | 0.916 | 0.71 |
| GBM | RUN5 | PA4 | 0.907 | 0.677 |
| GBM | RUN5 | PA5 | 0.898 | 0.666 |
| CTA | RUN1 | PA1 | 0.855 | 0.653 |
| CTA | RUN1 | PA2 | 0.868 | 0.693 |
| CTA | RUN1 | PA3 | 0.86 | 0.673 |
| CTA | RUN1 | PA4 | 0.817 | 0.633 |
| CTA | RUN1 | PA5 | 0.903 | 0.707 |
| CTA | RUN2 | PA1 | 0.873 | 0.669 |
| CTA | RUN2 | PA2 | 0.869 | 0.705 |
| CTA | RUN2 | PA3 | 0.875 | 0.685 |
| CTA | RUN2 | PA4 | 0.848 | 0.633 |
| CTA | RUN2 | PA5 | 0.885 | 0.711 |
| CTA | RUN3 | PA1 | 0.889 | 0.702 |
| CTA | RUN3 | PA2 | 0.865 | 0.689 |
| CTA | RUN3 | PA3 | 0.827 | 0.653 |
| CTA | RUN3 | PA4 | 0.796 | 0.593 |
| CTA | RUN3 | PA5 | 0.839 | 0.659 |
| CTA | RUN4 | PA1 | 0.833 | 0.617 |
| CTA | RUN4 | PA2 | 0.878 | 0.709 |
| CTA | RUN4 | PA3 | 0.9 | 0.734 |
| CTA | RUN4 | PA4 | 0.815 | 0.629 |
| CTA | RUN4 | PA5 | 0.858 | 0.711 |
| CTA | RUN5 | PA1 | 0.895 | 0.706 |
| CTA | RUN5 | PA2 | 0.858 | 0.68 |
| CTA | RUN5 | PA3 | 0.837 | 0.681 |
| CTA | RUN5 | PA4 | 0.798 | 0.597 |
| CTA | RUN5 | PA5 | 0.858 | 0.642 |
| ANN | RUN1 | PA1 | 0.881 | 0.665 |
| ANN | RUN1 | PA2 | 0.866 | 0.676 |
| ANN | RUN1 | PA3 | 0.861 | 0.661 |
| ANN | RUN1 | PA4 | 0.859 | 0.641 |
| ANN | RUN1 | PA5 | 0.878 | 0.69 |
| ANN | RUN2 | PA1 | 0.859 | 0.649 |
| ANN | RUN2 | PA2 | 0.865 | 0.656 |
| ANN | RUN2 | PA3 | 0.867 | 0.645 |
| ANN | RUN2 | PA4 | 0.869 | 0.637 |
| ANN | RUN2 | PA5 | 0.889 | 0.711 |
| ANN | RUN3 | PA1 | 0.876 | 0.613 |
| ANN | RUN3 | PA2 | 0.859 | 0.636 |
| ANN | RUN3 | PA3 | 0.815 | 0.556 |
| ANN | RUN3 | PA4 | 0.776 | 0.552 |
| ANN | RUN3 | PA5 | 0.835 | 0.646 |
| ANN | RUN4 | PA1 | 0.823 | 0.581 |
| ANN | RUN4 | PA2 | 0.862 | 0.705 |
| ANN | RUN4 | PA3 | 0.893 | 0.69 |
| ANN | RUN4 | PA4 | 0.867 | 0.629 |
| ANN | RUN4 | PA5 | 0.882 | 0.687 |
| ANN | RUN5 | PA1 | 0.889 | 0.677 |
| ANN | RUN5 | PA2 | 0.848 | 0.66 |
| ANN | RUN5 | PA3 | 0.856 | 0.685 |
| ANN | RUN5 | PA4 | 0.794 | 0.524 |
| ANN | RUN5 | PA5 | 0.846 | 0.622 |
| SRE | RUN1 | PA1 | 0.665 | 0.331 |
| SRE | RUN1 | PA2 | 0.667 | 0.334 |
| SRE | RUN1 | PA3 | 0.663 | 0.327 |
| SRE | RUN1 | PA4 | 0.663 | 0.327 |
| SRE | RUN1 | PA5 | 0.696 | 0.392 |
| SRE | RUN2 | PA1 | 0.702 | 0.403 |
| SRE | RUN2 | PA2 | 0.671 | 0.342 |
| SRE | RUN2 | PA3 | 0.696 | 0.391 |
| SRE | RUN2 | PA4 | 0.685 | 0.371 |
| SRE | RUN2 | PA5 | 0.71 | 0.42 |
| SRE | RUN3 | PA1 | 0.69 | 0.379 |
| SRE | RUN3 | PA2 | 0.681 | 0.362 |
| SRE | RUN3 | PA3 | 0.696 | 0.391 |
| SRE | RUN3 | PA4 | 0.698 | 0.395 |
| SRE | RUN3 | PA5 | 0.662 | 0.323 |
| SRE | RUN4 | PA1 | 0.675 | 0.351 |
| SRE | RUN4 | PA2 | 0.695 | 0.391 |
| SRE | RUN4 | PA3 | 0.681 | 0.363 |
| SRE | RUN4 | PA4 | 0.702 | 0.403 |
| SRE | RUN4 | PA5 | 0.682 | 0.363 |
| SRE | RUN5 | PA1 | 0.667 | 0.335 |
| SRE | RUN5 | PA2 | 0.677 | 0.354 |
| SRE | RUN5 | PA3 | 0.704 | 0.407 |
| SRE | RUN5 | PA4 | 0.671 | 0.343 |
| SRE | RUN5 | PA5 | 0.66 | 0.319 |
| FDA | RUN1 | PA1 | 0.908 | 0.698 |
| FDA | RUN1 | PA2 | 0.887 | 0.685 |
| FDA | RUN1 | PA3 | 0.88 | 0.677 |
| FDA | RUN1 | PA4 | 0.869 | 0.641 |
| FDA | RUN1 | PA5 | 0.896 | 0.719 |
| FDA | RUN2 | PA1 | 0.902 | 0.694 |
| FDA | RUN2 | PA2 | 0.89 | 0.689 |
| FDA | RUN2 | PA3 | 0.89 | 0.69 |
| FDA | RUN2 | PA4 | 0.888 | 0.681 |
| FDA | RUN2 | PA5 | 0.891 | 0.751 |
| FDA | RUN3 | PA1 | 0.909 | 0.698 |
| FDA | RUN3 | PA2 | 0.897 | 0.713 |
| FDA | RUN3 | PA3 | 0.902 | 0.71 |
| FDA | RUN3 | PA4 | 0.889 | 0.653 |
| FDA | RUN3 | PA5 | 0.881 | 0.663 |
| FDA | RUN4 | PA1 | 0.883 | 0.633 |
| FDA | RUN4 | PA2 | 0.92 | 0.717 |
| FDA | RUN4 | PA3 | 0.926 | 0.734 |
| FDA | RUN4 | PA4 | 0.89 | 0.69 |
| FDA | RUN4 | PA5 | 0.923 | 0.715 |
| FDA | RUN5 | PA1 | 0.919 | 0.706 |
| FDA | RUN5 | PA2 | 0.892 | 0.717 |
| FDA | RUN5 | PA3 | 0.902 | 0.738 |
| FDA | RUN5 | PA4 | 0.88 | 0.681 |
| FDA | RUN5 | PA5 | 0.882 | 0.642 |
| MARS | RUN1 | PA1 | 0.912 | 0.718 |
| MARS | RUN1 | PA2 | 0.901 | 0.705 |
| MARS | RUN1 | PA3 | 0.89 | 0.69 |
| MARS | RUN1 | PA4 | 0.88 | 0.661 |
| MARS | RUN1 | PA5 | 0.907 | 0.711 |
| MARS | RUN2 | PA1 | 0.907 | 0.685 |
| MARS | RUN2 | PA2 | 0.897 | 0.697 |
| MARS | RUN2 | PA3 | 0.897 | 0.722 |
| MARS | RUN2 | PA4 | 0.897 | 0.661 |
| MARS | RUN2 | PA5 | 0.902 | 0.727 |
| MARS | RUN3 | PA1 | 0.909 | 0.706 |
| MARS | RUN3 | PA2 | 0.895 | 0.717 |
| MARS | RUN3 | PA3 | 0.902 | 0.698 |
| MARS | RUN3 | PA4 | 0.887 | 0.629 |
| MARS | RUN3 | PA5 | 0.887 | 0.67 |
| MARS | RUN4 | PA1 | 0.88 | 0.653 |
| MARS | RUN4 | PA2 | 0.911 | 0.697 |
| MARS | RUN4 | PA3 | 0.925 | 0.746 |
| MARS | RUN4 | PA4 | 0.896 | 0.718 |
| MARS | RUN4 | PA5 | 0.918 | 0.743 |
| MARS | RUN5 | PA1 | 0.914 | 0.75 |
| MARS | RUN5 | PA2 | 0.891 | 0.709 |
| MARS | RUN5 | PA3 | 0.909 | 0.698 |
| MARS | RUN5 | PA4 | 0.899 | 0.653 |
| MARS | RUN5 | PA5 | 0.886 | 0.65 |
| RF | RUN1 | PA1 | 0.92 | 0.718 |
| RF | RUN1 | PA2 | 0.913 | 0.737 |
| RF | RUN1 | PA3 | 0.89 | 0.722 |
| RF | RUN1 | PA4 | 0.9 | 0.665 |
| RF | RUN1 | PA5 | 0.921 | 0.763 |
| RF | RUN2 | PA1 | 0.909 | 0.722 |
| RF | RUN2 | PA2 | 0.913 | 0.757 |
| RF | RUN2 | PA3 | 0.902 | 0.75 |
| RF | RUN2 | PA4 | 0.893 | 0.694 |
| RF | RUN2 | PA5 | 0.913 | 0.763 |
| RF | RUN3 | PA1 | 0.916 | 0.734 |
| RF | RUN3 | PA2 | 0.914 | 0.741 |
| RF | RUN3 | PA3 | 0.919 | 0.746 |
| RF | RUN3 | PA4 | 0.89 | 0.673 |
| RF | RUN3 | PA5 | 0.894 | 0.687 |
| RF | RUN4 | PA1 | 0.888 | 0.677 |
| RF | RUN4 | PA2 | 0.913 | 0.733 |
| RF | RUN4 | PA3 | 0.931 | 0.766 |
| RF | RUN4 | PA4 | 0.899 | 0.673 |
| RF | RUN4 | PA5 | 0.921 | 0.735 |
| RF | RUN5 | PA1 | 0.921 | 0.734 |
| RF | RUN5 | PA2 | 0.897 | 0.721 |
| RF | RUN5 | PA3 | 0.917 | 0.734 |
| RF | RUN5 | PA4 | 0.894 | 0.685 |
| RF | RUN5 | PA5 | 0.908 | 0.682 |

**(ANN: Artificial Neural Networks, CTA: Classification Tree Analysis, FDA: Flexible Discriminant Analysis, GBM: Generalized Boosting Models, GLM: Generalized Linear Models, MARS: Multivariate Adaptive Regression Splines, RF: Random Forest, SRE: Surface Range Envelop)**

**Table S2. Accuracy metrics (AUC and TSS) of individual species distribution models based on scientific survey data. Model refers to the specific individual model, Run indicates the number of model runs, and PA denotes the number of pseudo-absence replicates.**

| Model | Run | PA | AUC | TSS |
| --- | --- | --- | --- | --- |
| GLM | RUN1 | PA1 | 0.91 | 0.663 |
| GLM | RUN1 | PA2 | 0.9 | 0.679 |
| GLM | RUN1 | PA3 | 0.921 | 0.797 |
| GLM | RUN1 | PA4 | 0.932 | 0.715 |
| GLM | RUN1 | PA5 | 0.912 | 0.733 |
| GLM | RUN2 | PA1 | 0.872 | 0.692 |
| GLM | RUN2 | PA2 | 0.882 | 0.594 |
| GLM | RUN2 | PA3 | 0.946 | 0.765 |
| GLM | RUN2 | PA4 | 0.914 | 0.695 |
| GLM | RUN2 | PA5 | 0.907 | 0.717 |
| GLM | RUN3 | PA1 | 0.89 | 0.728 |
| GLM | RUN3 | PA2 | 0.896 | 0.662 |
| GLM | RUN3 | PA3 | 0.905 | 0.678 |
| GLM | RUN3 | PA4 | 0.887 | 0.679 |
| GLM | RUN3 | PA5 | 0.964 | 0.783 |
| GLM | RUN4 | PA1 | 0.898 | 0.726 |
| GLM | RUN4 | PA2 | 0.909 | 0.682 |
| GLM | RUN4 | PA3 | 0.921 | 0.746 |
| GLM | RUN4 | PA4 | 0.913 | 0.746 |
| GLM | RUN4 | PA5 | 0.945 | 0.817 |
| GLM | RUN5 | PA1 | 0.9 | 0.707 |
| GLM | RUN5 | PA2 | 0.896 | 0.648 |
| GLM | RUN5 | PA3 | 0.84 | 0.662 |
| GLM | RUN5 | PA4 | 0.882 | 0.613 |
| GLM | RUN5 | PA5 | 0.916 | 0.717 |
| GBM | RUN1 | PA1 | 0.929 | 0.713 |
| GBM | RUN1 | PA2 | 0.929 | 0.729 |
| GBM | RUN1 | PA3 | 0.955 | 0.798 |
| GBM | RUN1 | PA4 | 0.951 | 0.765 |
| GBM | RUN1 | PA5 | 0.94 | 0.733 |
| GBM | RUN2 | PA1 | 0.908 | 0.725 |
| GBM | RUN2 | PA2 | 0.919 | 0.682 |
| GBM | RUN2 | PA3 | 0.945 | 0.749 |
| GBM | RUN2 | PA4 | 0.918 | 0.729 |
| GBM | RUN2 | PA5 | 0.925 | 0.683 |
| GBM | RUN3 | PA1 | 0.913 | 0.745 |
| GBM | RUN3 | PA2 | 0.923 | 0.696 |
| GBM | RUN3 | PA3 | 0.903 | 0.662 |
| GBM | RUN3 | PA4 | 0.911 | 0.679 |
| GBM | RUN3 | PA5 | 0.975 | 0.867 |
| GBM | RUN4 | PA1 | 0.928 | 0.745 |
| GBM | RUN4 | PA2 | 0.92 | 0.697 |
| GBM | RUN4 | PA3 | 0.936 | 0.731 |
| GBM | RUN4 | PA4 | 0.939 | 0.798 |
| GBM | RUN4 | PA5 | 0.959 | 0.817 |
| GBM | RUN5 | PA1 | 0.912 | 0.761 |
| GBM | RUN5 | PA2 | 0.892 | 0.665 |
| GBM | RUN5 | PA3 | 0.907 | 0.679 |
| GBM | RUN5 | PA4 | 0.916 | 0.714 |
| GBM | RUN5 | PA5 | 0.937 | 0.717 |
| CTA | RUN1 | PA1 | 0.811 | 0.622 |
| CTA | RUN1 | PA2 | 0.893 | 0.698 |
| CTA | RUN1 | PA3 | 0.911 | 0.73 |
| CTA | RUN1 | PA4 | 0.839 | 0.679 |
| CTA | RUN1 | PA5 | 0.853 | 0.667 |
| CTA | RUN2 | PA1 | 0.836 | 0.658 |
| CTA | RUN2 | PA2 | 0.804 | 0.628 |
| CTA | RUN2 | PA3 | 0.869 | 0.664 |
| CTA | RUN2 | PA4 | 0.839 | 0.678 |
| CTA | RUN2 | PA5 | 0.839 | 0.617 |
| CTA | RUN3 | PA1 | 0.87 | 0.693 |
| CTA | RUN3 | PA2 | 0.84 | 0.58 |
| CTA | RUN3 | PA3 | 0.856 | 0.631 |
| CTA | RUN3 | PA4 | 0.797 | 0.594 |
| CTA | RUN3 | PA5 | 0.902 | 0.75 |
| CTA | RUN4 | PA1 | 0.858 | 0.657 |
| CTA | RUN4 | PA2 | 0.797 | 0.594 |
| CTA | RUN4 | PA3 | 0.833 | 0.647 |
| CTA | RUN4 | PA4 | 0.903 | 0.731 |
| CTA | RUN4 | PA5 | 0.931 | 0.8 |
| CTA | RUN5 | PA1 | 0.912 | 0.713 |
| CTA | RUN5 | PA2 | 0.854 | 0.597 |
| CTA | RUN5 | PA3 | 0.826 | 0.613 |
| CTA | RUN5 | PA4 | 0.821 | 0.596 |
| CTA | RUN5 | PA5 | 0.897 | 0.667 |
| ANN | RUN1 | PA1 | 0.895 | 0.743 |
| ANN | RUN1 | PA2 | 0.919 | 0.697 |
| ANN | RUN1 | PA3 | 0.827 | 0.593 |
| ANN | RUN1 | PA4 | 0.92 | 0.679 |
| ANN | RUN1 | PA5 | 0.868 | 0.667 |
| ANN | RUN2 | PA1 | 0.878 | 0.66 |
| ANN | RUN2 | PA2 | 0.885 | 0.629 |
| ANN | RUN2 | PA3 | 0.876 | 0.628 |
| ANN | RUN2 | PA4 | 0.834 | 0.644 |
| ANN | RUN2 | PA5 | 0.839 | 0.583 |
| ANN | RUN3 | PA1 | 0.836 | 0.576 |
| ANN | RUN3 | PA2 | 0.826 | 0.579 |
| ANN | RUN3 | PA3 | 0.852 | 0.531 |
| ANN | RUN3 | PA4 | 0.862 | 0.713 |
| ANN | RUN3 | PA5 | 0.828 | 0.567 |
| ANN | RUN4 | PA1 | 0.849 | 0.605 |
| ANN | RUN4 | PA2 | 0.835 | 0.614 |
| ANN | RUN4 | PA3 | 0.869 | 0.58 |
| ANN | RUN4 | PA4 | 0.848 | 0.629 |
| ANN | RUN4 | PA5 | 0.835 | 0.683 |
| ANN | RUN5 | PA1 | 0.864 | 0.624 |
| ANN | RUN5 | PA2 | 0.798 | 0.529 |
| ANN | RUN5 | PA3 | 0.797 | 0.53 |
| ANN | RUN5 | PA4 | 0.822 | 0.562 |
| ANN | RUN5 | PA5 | 0.863 | 0.7 |
| SRE | RUN1 | PA1 | 0.682 | 0.365 |
| SRE | RUN1 | PA2 | 0.633 | 0.265 |
| SRE | RUN1 | PA3 | 0.692 | 0.383 |
| SRE | RUN1 | PA4 | 0.625 | 0.249 |
| SRE | RUN1 | PA5 | 0.667 | 0.333 |
| SRE | RUN2 | PA1 | 0.707 | 0.415 |
| SRE | RUN2 | PA2 | 0.699 | 0.398 |
| SRE | RUN2 | PA3 | 0.733 | 0.467 |
| SRE | RUN2 | PA4 | 0.666 | 0.332 |
| SRE | RUN2 | PA5 | 0.675 | 0.35 |
| SRE | RUN3 | PA1 | 0.616 | 0.231 |
| SRE | RUN3 | PA2 | 0.649 | 0.299 |
| SRE | RUN3 | PA3 | 0.7 | 0.399 |
| SRE | RUN3 | PA4 | 0.649 | 0.298 |
| SRE | RUN3 | PA5 | 0.783 | 0.567 |
| SRE | RUN4 | PA1 | 0.675 | 0.349 |
| SRE | RUN4 | PA2 | 0.7 | 0.399 |
| SRE | RUN4 | PA3 | 0.675 | 0.349 |
| SRE | RUN4 | PA4 | 0.7 | 0.399 |
| SRE | RUN4 | PA5 | 0.742 | 0.483 |
| SRE | RUN5 | PA1 | 0.641 | 0.282 |
| SRE | RUN5 | PA2 | 0.724 | 0.449 |
| SRE | RUN5 | PA3 | 0.708 | 0.416 |
| SRE | RUN5 | PA4 | 0.616 | 0.231 |
| SRE | RUN5 | PA5 | 0.758 | 0.517 |
| FDA | RUN1 | PA1 | 0.869 | 0.692 |
| FDA | RUN1 | PA2 | 0.898 | 0.648 |
| FDA | RUN1 | PA3 | 0.925 | 0.746 |
| FDA | RUN1 | PA4 | 0.894 | 0.731 |
| FDA | RUN1 | PA5 | 0.912 | 0.767 |
| FDA | RUN2 | PA1 | 0.863 | 0.625 |
| FDA | RUN2 | PA2 | 0.903 | 0.713 |
| FDA | RUN2 | PA3 | 0.909 | 0.697 |
| FDA | RUN2 | PA4 | 0.895 | 0.664 |
| FDA | RUN2 | PA5 | 0.918 | 0.767 |
| FDA | RUN3 | PA1 | 0.85 | 0.709 |
| FDA | RUN3 | PA2 | 0.913 | 0.748 |
| FDA | RUN3 | PA3 | 0.892 | 0.697 |
| FDA | RUN3 | PA4 | 0.849 | 0.678 |
| FDA | RUN3 | PA5 | 0.948 | 0.817 |
| FDA | RUN4 | PA1 | 0.912 | 0.728 |
| FDA | RUN4 | PA2 | 0.877 | 0.714 |
| FDA | RUN4 | PA3 | 0.9 | 0.714 |
| FDA | RUN4 | PA4 | 0.89 | 0.696 |
| FDA | RUN4 | PA5 | 0.952 | 0.817 |
| FDA | RUN5 | PA1 | 0.886 | 0.708 |
| FDA | RUN5 | PA2 | 0.878 | 0.647 |
| FDA | RUN5 | PA3 | 0.87 | 0.679 |
| FDA | RUN5 | PA4 | 0.869 | 0.663 |
| FDA | RUN5 | PA5 | 0.909 | 0.683 |
| MARS | RUN1 | PA1 | 0.842 | 0.676 |
| MARS | RUN1 | PA2 | 0.913 | 0.714 |
| MARS | RUN1 | PA3 | 0.947 | 0.747 |
| MARS | RUN1 | PA4 | 0.908 | 0.765 |
| MARS | RUN1 | PA5 | 0.921 | 0.717 |
| MARS | RUN2 | PA1 | 0.885 | 0.659 |
| MARS | RUN2 | PA2 | 0.91 | 0.695 |
| MARS | RUN2 | PA3 | 0.891 | 0.628 |
| MARS | RUN2 | PA4 | 0.915 | 0.73 |
| MARS | RUN2 | PA5 | 0.917 | 0.717 |
| MARS | RUN3 | PA1 | 0.902 | 0.712 |
| MARS | RUN3 | PA2 | 0.879 | 0.748 |
| MARS | RUN3 | PA3 | 0.891 | 0.695 |
| MARS | RUN3 | PA4 | 0.87 | 0.661 |
| MARS | RUN3 | PA5 | 0.966 | 0.833 |
| MARS | RUN4 | PA1 | 0.921 | 0.777 |
| MARS | RUN4 | PA2 | 0.847 | 0.629 |
| MARS | RUN4 | PA3 | 0.875 | 0.662 |
| MARS | RUN4 | PA4 | 0.9 | 0.646 |
| MARS | RUN4 | PA5 | 0.941 | 0.883 |
| MARS | RUN5 | PA1 | 0.892 | 0.694 |
| MARS | RUN5 | PA2 | 0.897 | 0.644 |
| MARS | RUN5 | PA3 | 0.899 | 0.697 |
| MARS | RUN5 | PA4 | 0.88 | 0.698 |
| MARS | RUN5 | PA5 | 0.873 | 0.683 |
| RF | RUN1 | PA1 | 0.936 | 0.747 |
| RF | RUN1 | PA2 | 0.944 | 0.732 |
| RF | RUN1 | PA3 | 0.965 | 0.816 |
| RF | RUN1 | PA4 | 0.952 | 0.747 |
| RF | RUN1 | PA5 | 0.945 | 0.75 |
| RF | RUN2 | PA1 | 0.912 | 0.724 |
| RF | RUN2 | PA2 | 0.931 | 0.749 |
| RF | RUN2 | PA3 | 0.947 | 0.75 |
| RF | RUN2 | PA4 | 0.925 | 0.712 |
| RF | RUN2 | PA5 | 0.946 | 0.75 |
| RF | RUN3 | PA1 | 0.92 | 0.728 |
| RF | RUN3 | PA2 | 0.925 | 0.747 |
| RF | RUN3 | PA3 | 0.913 | 0.713 |
| RF | RUN3 | PA4 | 0.923 | 0.747 |
| RF | RUN3 | PA5 | 0.975 | 0.85 |
| RF | RUN4 | PA1 | 0.932 | 0.744 |
| RF | RUN4 | PA2 | 0.919 | 0.699 |
| RF | RUN4 | PA3 | 0.946 | 0.764 |
| RF | RUN4 | PA4 | 0.936 | 0.781 |
| RF | RUN4 | PA5 | 0.96 | 0.833 |
| RF | RUN5 | PA1 | 0.917 | 0.745 |
| RF | RUN5 | PA2 | 0.892 | 0.63 |
| RF | RUN5 | PA3 | 0.907 | 0.696 |
| RF | RUN5 | PA4 | 0.916 | 0.665 |
| RF | RUN5 | PA5 | 0.944 | 0.733 |
